# Cytokinin Transporters: Multisite Players in Cytokinin Homeostasis and Signal Distribution

**DOI:** 10.3389/fpls.2019.00693

**Published:** 2019-05-31

**Authors:** Chang-Jun Liu, Yunjun Zhao, Kewei Zhang

**Affiliations:** ^1^Department of Biology, Brookhaven National Laboratory, Upton, NY, United States; ^2^Department of Biology, College of Chemistry and Life Sciences, Zhejiang Normal University, Jinhua, China

**Keywords:** cytokinin, ATP-binding cassette transporter, purine permease, equilibrative nucleoside transporter, isopentenyl adenine, *trans*-zeatin

## Abstract

Cytokinins (CKs) are a group of mobile adenine derivatives that act as chemical signals regulating a variety of biological processes implicated in plant development and stress responses. Their synthesis, homeostasis, and signaling perception evoke complicated intracellular traffic, intercellular movement, and in short- and long-distance translocation. Over nearly two decades, subsets of membrane transporters have been recognized and implicated in the transport of CKs as well as the related adenylates. In this review, we aim to recapitulate the key progresses in exploration of the transporter proteins involved in cytokinin traffic and translocation, discuss their functional implications in the cytokinin-mediated paracrine and long-distance communication, and highlight some knowledge gaps and open issues toward comprehensively understanding the molecular mechanism of membrane transporters in controlling spatiotemporal distribution of cytokinin species.

## Introduction

Cytokinins (CKs) are the mobile adenine derivatives that carry *N*^6^-linked isopentenyl or aromatic side chains. They serve as hormonal signals functioning in a myriad of biological processes, such as cell division and differentiation, seed germination, apical dominance, leaf senescence, root growth, branching and nodulation, nutrient homeostasis, and stress responses (Mok and Mok, 2001; Sakakibara, 2006; Werner and Schmulling, 2009; Kieber and Schaller, 2018). The majority of naturally occurring CKs *in planta* are the *N*^6^-isopentenyl conjugated adenine derivatives, in addition to a small amount of *N*^6^-aromatic CK species. The isoprenoid CKs chemically differ in their side chain structures, including the hydroxylation at the side chain terminus, the stereo-isomeric position, and the hydrocarbon chain saturation status. Correspondingly, they are classified into four main categories: *N*^6^-(Δ^2^-isopentenyl) adenine (iP), *trans*-zeatin (tZ), *cis*-zeatin (cZ), and dihydrozeatin (DHZ) (Sakakibara, 2006). Structural variations of the side chain may confer substantial differences in chemical properties of CK species, especially for their lipophilicity/hydrophobicity. Predictably, the iP-type CKs that lack hydroxyl moiety at their *N*^6^-linked side chains would possess the highest hydrophobicity compared to the others.

The isoprenoid CKs are generated through *N*-prenylation of adenosine 5′-phosphates (AMP, ADP, or ATP) or tRNAs, which is catalyzed by adenosine 5′-phosphate isopentenyltransferase (IPT) (Kakimoto, 2001; Takei et al., 2001, 2004a; Miyawaki et al., 2006). The resulting product isopentenyl adenosine 5′-phosphates, i.e., iP nucleotides, are then converted to tZ derivatives by *trans*-hydroxylases, the cytochrome P450 enzymes CYP735A1 and CYP735A2 (Takei et al., 2004b). Both iP- and tZ- nucleotides can be activated by phosphoribohydrolase LONELY GUY (LOG), yielding free CK bases (Kurakawa et al., 2007; Ueda et al., 2007). Alternatively, the formation of free CK bases may potentially undergo a two-step sequential dephosphorylation and deribosylation process. However, only a nucleoside *N*-ribohydrolase that mediates deribosylation reaction has been recognized so far (Jung et al., 2009; Kopecna et al., 2013). The enzymes involved in the two-step conversion *in planta* remain to be further identified.

Cytokinins are synthesized in a number of different cell types in both roots and shoots, and cross-talk with other phytohormones, particularly auxins, to regulate plant growth and development (Sakakibara, 2006). As chemical signals, CKs mediate both local and long-distance communications, and are transported either in short distance among neighboring cells or as acropetal and basipetal messengers translocated in long distance between roots and shoots. In Arabidopsis, tZ- and iP-type of CK species are confirmed as the active forms in respect to their specific recognition by three sensor histidine kinases, AHK2, AHK3, and CRE1/AHK4 (Inoue et al., 2001; Kakimoto, 2003; Romanov et al., 2006; Lomin et al., 2015). The tZ-type CKs are mainly synthesized in roots and transported apoplastically to shoots, which promote the growth of the above-ground parts of the plant (Beveridge et al., 1997; Hirose et al., 2008). In contrast, the iP- and cZ type CKs are the major forms found in phloem and are translocated rootward to transmit messages from shoots to roots (Corbesier et al., 2003; Hirose et al., 2008). The shoot-borne iP-type CKs have been suggested to serve as a signal of nitrogen satiety, regulating root architecture, suppressing nitrogen uptake in the root, and/or modulating nodulation (Sasaki et al., 2014). In addition to nutritional signaling, the shoot-derived iP-type CKs also regulate root development by modulating polar auxin transport and vascular patterning in the root meristem (Bishopp et al., 2011). Interestingly, although the iP-type CKs are considered to be synthesized throughout the whole plant body of Arabidopsis (Miyawaki et al., 2004, 2006; Takei et al., 2004a), a couple of recent studies reveal that iP ribosides can be predominantly synthesized in the roots of young tobacco and Arabidopsis seedlings (Zd’arska et al., 2013; Gelova et al., 2018), which implicate that a shootward transport of iPR via phloem could also be possible, although its underlying mechanism and related physiological functions remain to be explored.

**FIGURE 1:**
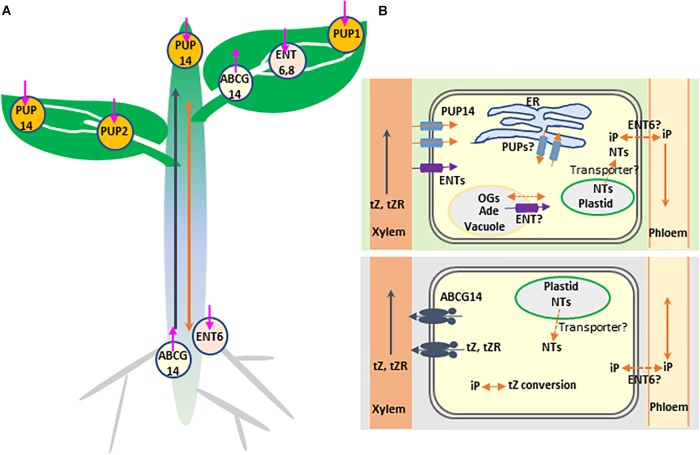
Schematic representation of transporter-mediated long- and short-distance movement of cytokinins in *Arabidopsis thaliana*. **(A)** Involvement of transporters in cytokinin distribution. The determined or the speculated functional sites of cytokinin transporters, based on their expression patterns (see Table 1 for further details), are mapped on the different tissues. The three types of transporters are indicated with distinctly colored circles. The orientation of the transport is indicated by an arrow pointing inside (influx) or outside (efflux) the circle symbol. Long-distance transport of tZ-type cytokinins from roots to photosynthetic tissues through xylem and the rootward and the hypothetic shootward translocation of iP-type cytokinins through phloem are indicated with blue and orange arrows, respectively. **(B)** Involvement of transporters in the inter-and intra-cellular movements of cytokinins in root (bottom) and shoot (top) cells. The plastid-synthesized iP nucleotides (NTs) may be delivered to the cytoplasm, nucleus, or even endoplasmic reticulum (ER) via transporter activity. ABCG14 as an efflux pump transports tZ-type cytokinins from cytoplasm to apoplast (xylem vessel). PUP14 and ENTs act as importers to take up apoplastic free bases or nucleosides of cytokinins, respectively, into cytoplasm. The ER localized PUP members may involve in sequestering cytokinin species to or out ER lumen. ENT transporter perhaps delivers vacuolar adenosine (Ade) to cytosol; meanwhile, the unidentified transporters might be responsible for the vacuolar sequestration of reversible cytokinin glucosides (OGs). Finally, transport of iP-type cytokinin species in or out of the cells of their synthesis and the signaling target sites as well as phloem may also require transporter proteins; ENT6 was considered as one of the candidates.

**Table 1:** The identified and/or putative cytokinin transporters^∗^.

Transporter family	Name	Substrates (organism for detection)	Physiological functions in plant	Subcellular localization	Expression pattern	References
**ENT**	AtENT1	Adenosine in yeast	Unknown	Plasma membrane or vacuolar membrane		Li and Wang, 2000; Sun et al., 2005; Jaquinod et al., 2007
	AtENT8/SOI33	iPR in seedlings	Reduced sensitivity of *soi33-1* to iPR; Overexpression increases sensitivity to iPR	Unknown	Seedling, hypocotyls, flowers, midribs, abscission zones, siliques	Sun et al., 2005
	AtENT3	iPR in seedlings; adenosine in yeast; nucleoside in Xenopus oocytes	Reduced uptake efficiency of nucleoside-type CK (iPR), seedlings	Unknown	Unknown	Li et al., 2003; Sun et al., 2005
	AtENT6	iPR, tZR and adenosine in yeast	Unknown	Plasma membrane	Vascular tissues of root, leaf and flower, and cotyledons, stomata	Wormit et al., 2004; Hirose et al., 2008
	OsENT2	iPR, tZR, adenosine and uridine in yeast	Unknown	Unknown	Vascular tissues of leaf and root	Hirose et al., 2005
**PUP**	AtPUP1	Hypoxanthine, zeatin, kinetin, caffeine in yeast (proton-gradient dependent symporter)	Unknown	Unknown	Leaf mesophyll cells, stems, flowers, siliques, and hydathodes, stigma	Gillissen et al., 2000; Bürkle et al., 2003
	AtPUP2	Adenine, tZ, cZ, iPA, kinetin, BAP and tZR in yeast (proton-gradient dependent symporter)	Unknown	Unknown	Vascular tissues of leaf, stem, particularly in phloem, pollen	Bürkle et al., 2003
	AtPUP14	tZ, iPA, BA in protoplast and microsomes of plant. (ATP- dependent)	CK signaling, morphogenesis in embryos, roots and the shoot apical meristem	Plasma membrane	Various organ and tissues, including seedlings, embryos and mesophyll cells	Zürcher et al., 2016
	OsPUP7	Caffeine in yeast	Plant height, seed size and flowering time. Higher iP and iPR levels in mutant plant	Endoplasmic reticulum	Vascular bundle of roots, culms and leaves, hull vein and flower, stigma, style, stamens	Qi and Xiong, 2013; Kirschner et al., 2018; Xiao et al., 2018
	OsPUP4/BG3	Unknown	Seed size	Plasma membrane	Parenchyma cells near vascular tissue	Xiao et al., 2018
**ABCG**	AtABCG14	tZ-type CKs	Reduced root-to-shoot translocation in mutant. Shoot and root growth, nitrogen signaling	Plasma membrane	Vascular tissue in root (mainly) and shoot, leaf midribs, veins, mature anthers	Ko et al., 2014; Zhang et al., 2014; Poitout et al., 2018

*^∗^iPA or iP, isopentenyladenine; iPR, isopentenyladenosine; BAP or BA, benzylaminopurine or benzyladenine; tZ, trans-zeatin; tZR, trans-zeatin riboside.*

As mobile signals, CKs biosynthesis, metabolism, distribution and perception evoke considerable intra- and inter-cellular movement and translocation. However, compared with the knowledge on transport and distribution of other plant growth and development related phytohormones, such as auxin, our understanding on molecular mechanisms of CK transport is just emerging. During the last two decades, three types of membrane transporters have been recognized and implicated in the CK transmembrane transport and intercellular translocation (Figure 1 and Table 1). These proteins include the subsets of purine permeases (PUPs) and equilibrative nucleoside transporters (ENTs), which act as influx carriers and are implicated in the transport of CK nucleobases or nucleosides, respectively (Figure 1 and Table 1), and the ATP-binding cassette transporter G subfamily member, ABCG14 in Arabidopsis, that acts as an efflux pump involved in long-distance acropetal translocation of the root-born CKs (Figure 1 and Table 1). Over the years, several excellent review articles have summarized the progress in understanding CK transport and in characterizing the related transporters (Hirose et al., 2008; Kudo et al., 2010; Borghi et al., 2015; Lacombe and Achard, 2016; Duran-Medina et al., 2017; Kang et al., 2017; Ko and Helariutta, 2017; Park et al., 2017). In this article, we aim to recapitulate the advances in identifying and characterizing the membrane transporters involved in short- and long-distance translocation of CKs as well as the related metabolites, to discuss their implicated functions in CK biosynthesis, homeostasis, and signal perception, and to tentatively point out some gaps and open questions toward fully understanding molecular mechanism of CK transport.

## Transport Proteins Potentially Involved in Intracellular Traffic of Cytokinin

Subcellular compartmentation is a key feature of eukaryotic cells to effectively organize their metabolic and signaling processes (Skalicky et al., 2018). At the site of synthesis, the formation, conversion and storage of CKs take place in different compartments of the cell. Correspondingly the key enzymes/proteins involved in the processes are located within different places (Skalicky et al., 2018). IPTs catalyze *N*-prenylation of adenine ribotides, which diverts both the plastidial and cytosolic sources of terpenoid building blocks, the five carbon units, isopentenyl diphosphate (IPP) and dimethylallyl diphosphate (DMAPP) to CK synthesis (Sakakibara, 2006). Among several IPTs identified in Arabidopsis, AtIPT1, 3, and 5 were proved the major players for iP- and tZ-type CKs formation. Their fluorescently tagged proteins were found to primarily localize to the chloroplasts of mesophyll cells (Kasahara et al., 2004). Interestingly, besides its plastidial localization, AtIPT3 can be farnesylated, which directs it to the nucleus. The subcellular localization of farnesylated and non-farnesylated protein is closely correlated with either iP-type or tZ-type CK biosynthesis (Galichet et al., 2008). By contrast, AtIPT2 and 9 catalyze isopentenylation of tRNA to provide a source for cZ-type CKs (Miyawaki et al., 2006). The AtIPT2-GFP fusion appeared within cytoplasm, indicating that AtIPT2 utilizes isoprenoid precursors synthetized via the mevalonate pathway in cytosol for cZ-type CK synthesis. Additionally, AtIPT7-GFP was observed in mitochondria (Kasahara et al., 2004). Different subcellular localization of IPTs implicates multiple sites occurrence of CK species.

After formation of iP nucleotides, their subsequent hydroxylation is catalyzed by cytochrome P450 monooxygenases CYP735A1 and CYP735A2, which yields tZ-type CKs (Takei et al., 2004b). Cytochrome P450s are the integral endoplasmic reticulum (ER)-resident proteins. Therefore, the hydroxylation reaction of CKs must take place on the surface of ER. In addition, the resulting CK nucleotides need to be dephosphoribosylated to become active free-base forms (Kurakawa et al., 2007). The responsible enzyme LOGs have been demonstrated to predominantly present in the nucleus and cytosol (Kuroha et al., 2009). Therefore, the translocation of iP nucleotides from plastid to cytoplasm, to nucleus, or even to ER is conceivable (Figure 1). However, until now there is no specific study elucidating how the CK biosynthetic intermediates are intracellularly sequestrated, and whether the specific transporters are required. An Arabidopsis plastidial membrane-localized nucleotide uniport carrier, Brittle 1, has been found to transport adenylates such as AMP, ADP, and ATP into the cytosol (Kirchberger et al., 2008). The gene encoding Brittle 1 primarily expressed in the root tips and the maturating or germinating pollens. Down-regulation of *Brittle 1* gene expression substantially retarded plant growth; but feeding adenosine complemented the growth defect of the mutant (Kirchberger et al., 2008). Giving the structural similarity of prenylated adenosine-5′-phosphate to the reported substrates of Brittle 1, it remains interesting to examine whether such a plastidial purine nucleotide exporter would have overlapped functions in delivering CK biosynthetic intermediates from plastid to cytoplasm.

The free CK bases within the cell can be reversibly glycosylated at their hydroxyl moieties of the *N*^6^-side chains (for tZ, cZ, and DHZ), or irreversibly glycosylated at *N*^7^- or *N*^9^- positions by cytosolic localized glycosyltransferases and sequestered into the vacuole for storage. The vacuolar stored CK glucosides can also be reconverted back to the active CK species via β-glucosidases (Brzobohaty et al., 1993) and released to cytosol. In addition, the free CK bases can also be reversibly inactivated into their nucleotide forms by enzymes in common with purine metabolism, such as adenine phosphoribosyltransferases, which appear to be cytosolic and act antagonistically to LOGs (Moffatt et al., 1991; Zhang et al., 2013). Such a complexity in subcellular compartmentation of CK *de novo* synthesis, bioconversion, storage and inactivation implicates the presence of a sophisticated intracellular CK transport network. Members of equilibrative nucleoside transporter (ENT) family have been demonstrated with broad substrate specificities, transporting both purine and pyrimidine nucleosides (Mohlmann et al., 2001; Wormit et al., 2004; Table 1). Eight members (AtENT1-8) are found in Arabidopsis and four (OsENT1-4) in rice (Li et al., 2003; Hirose et al., 2004, 2005). Among them AtENT1 was the first member with demonstrated adenosine transport activity by a complementation assay with Arabidopsis cDNAs in yeast cells deficient in synthesizing adenine (Mohlmann et al., 2001). Instead of the previously reported plasma membrane localization (Li and Wang, 2000), AtENT1 was re-verified to present on vacuolar membrane (Jaquinod et al., 2007; Bernard et al., 2011; Schulze et al., 2012). Overexpression of *AtENT1* reduced vacuolar contents of adenosine and 2’,3’-adenosine monophosphate, the RNA degradation products (Bernard et al., 2011). Conversely, down-regulation of *AtENT1* by RNAi resulted in a significant increase in vacuolar accumulation of both metabolites. Furthermore, upregulating AtENT1-mediated vacuolar export activity concurred with the increase of the cytosolic adenosine kinase activity (Bernard et al., 2011). These observations suggest that AtENT1 might participate in the export of vacuolar nucleosides and/or RNA breakdown products to cytosol, and the exported nucleosides may re-enter the salvage pathways of adenylates with actions of nucleoside kinases, hydrolases, and/or phosphoribosyltransferases (Bernard et al., 2011). Therefore, expectedly AtENT1 activity might connect to the cytosolic CK biosynthesis and CK homeostasis. Although it was reported that the null mutant line of *AtENT1* exhibited no substantial effect on the CK response (Sun et al., 2005), it is worthy to re-examine the functions of this transporter in CK metabolism and homeostasis by using more sensitive CK signaling read-out reporters.

## Transporters Responsible for Cytokinin Long Distance Translocation

After synthesis, CKs undergo intercellular movement and long-distance translocation from the sites of their synthesis to the target cells. Consistently, CKs are detected in the transduction systems phloem and xylem sap. In xylem sap, tZ, and tZR account for about 15 and 80% of CK species, respectively (Beveridge et al., 1997; Hirose et al., 2008; Kuroha et al., 2009; Osugi et al., 2017); whereas in phloem sap, the major forms are the iP- and cZ-type CKs (Corbesier et al., 2003; Hirose et al., 2008). The compartmentation of tZ- and iP-type of CKs in distinct vascular tissues suggest that plant has elaborated sophisticated selective transport mechanisms for distributing different types of CKs.

Translocation of the root-born tZ-type of CKs from roots to shoots requires an ABC transporter (Figure 1). A few years ago, we adopted a reverse genetics approach to systematically characterize the functions of ABCG subfamily proteins. We noticed that disruption of ABCG14 in Arabidopsis caused obvious morphological alterations, such as smaller inflorescences, smaller rosettes, slender stems with reduced number of vascular bundles, severely retarded primary root growth, and differential responses to the exogenously supplied phytohormones. Such developmental defects resembled those of the cytokinin biosynthetic mutants defective in CK signaling (Zhang et al., 2014). AtABCG14 expressed primarily in the pericycle and stele of roots. Profiling CKs in both roots and shoots of Arabidopsis seedling deficient in *abcg14* revealed a clear overaccumulation of the root-derived tZ- and DHZ-type CKs in the roots and reduced content in the shoots, indicating that disruption of AtABCG14 substantially inhibits the translocation and distribution of the root-synthesized CKs to the shoots. Interestingly, in contrast to the decline of both tZ- and DHZ-type CKs in the shoots, the iP-type CKs levels were slightly increased in both the shoots and roots of *abcg14*, so was the cZ-type CKs (Zhang et al., 2014). These data point out that (1) AtABCG14 participates in the long-distance translocation of the root-derived CKs. (2) AtABCG14 possesses profound substrate specificity and is able to discriminate the subtle structural variation of the isopentenyl side chain of CK species. (3) An intrinsic CK homeostatic mechanism occurs in plant cells that compensates the perturbation of CK allocation. Furthermore, by employing radiolabeled tZ in an *in planta* feeding and in the detached leaf assay with *ABCG14* overexpressing plants, we demonstrated that ABCG14, as a plasma membrane-localized protein, functioned as an efflux pump (Zhang et al., 2014). The data affirm that ABCG14 is a CK exporter responsible for loading the root-born tZ- and DHZ-type CKs into xylem vessels from xylary parenchyma cells. Coincidently, a couple of months later, Ko et al. (2014) reported a very similar study and finding, with additional reciprocal grafting experiments confirming the roles of AtABCG14 in the long-distance translocation of the root-synthesized CKs to the shoots (Ko et al., 2014). ABC transporter is an ATP-dependent primary transporter. Its activity consumes ATP yielding AMP, which directly links to the purine nucleotide metabolism. Therefore, it might have metabolic and regulatory advantages for plant to adopt this type of transporter to deliver CKs species in terms of the potential co-sharing and recycling of purine nucleotides, and more effective coordination and organization of energy flow, CKs synthesis and transport processes.

When *AtABCG14::GUS* was employed to examine gene expression, we noticed that in adult plant, *AtABCG14* was not only expressed in the roots but also in young rosette leaves, especially in the leaf midribs, veins and the nearby leaf cells. In addition, *AtABCG14* was also detected in mature anthers, and in siliques (Zhang et al., 2014; Table 1). The broad expression pattern of *AtABCG14* hints its possibility of more comprehensive functions than the involvement in xylem loading of root CKs. Speculatively, AtABCG14 may also reside on the cell membrane of the lignifying yet alive vascular cells near midribs or veins of young aerial tissues, potentially as an efflux pump responsible for the redistribution of CK species that are accumulated in those alive xylary cells (Figure 1), although currently it remains unclear how the CK species from xylem sap enrich in those cells. Alternatively, when AtABCG14 expresses in the leaf vein neighboring cells, it may also be possible to switch its transport direction to function as an importer taking up the xylem sap CKs to the cells. Although it is quite a rare case and mechanistically unclear, some ABC transporters seem pretty versatile in their flux pump direction. For example, AtABCB4, the transporter involved in auxin distribution, exhibited a substrate concentration-dependent switch from auxin influx to efflux when it was heterologously expressed in tobacco BY-2 cells, yeast, or HeLa cells (Terasaka et al., 2005; Yang and Murphy, 2009; Kubes et al., 2012). It is unknown whether such a unique behavior occurs *in planta*. Clearly, more refined experimental evidences are required for corroborating the postulated dual roles of AtABCG14 in CK translocation and cellular distribution.

While ABCG14 mediates acropetal transport of the root-born tZs to the shoots, systemic transport of the shoot-derived CKs (primarily iP- and cZ-types) takes place in phloem and likely involves cell to cell translocation through symplastic connection (Figure 1). Plasmodesmata connects neighboring cells and forms a highway for the movement of endogenous CKs as well as other photosynthetic assimilates from the site of synthesis to phloem and from phloem to the target cells (Romanov et al., 2018). This symplastic process appears to have no requirement for the involvement of particular transporters. However, previous studies revealed that AtENT6 in Arabidopsis and OsENT2 in rice were likely involved in mediating the transport of adenosine and other nucleosides, including iP riboside (iPR) (Hirose et al., 2005, 2008; Table 1). Comparably, the affinity of ENTs to iPR was much higher than that to tZR (Hirose et al., 2005, 2008). Moreover, AtENT6 predominantly expressed in the vascular tissues of rosette leaves, flowers, cotyledons and roots (Hirose et al., 2008), while OsENT2 expressed primarily in rice leaf vascular bundles and phloem tissues (Hirose et al., 2005; Table 1). These data led to the speculation that ENTs may be responsible at least in part for the selective transport of iP nucleosides in vascular tissues (Sakakibara, 2006). Considering the hydrophobic characteristics of iP species, another possibility is that the concentration-dependent free diffusion might also contribute to iPs’ spatial distribution and movement. This notion seems to be supported with the observation that the iP-type CKs abundantly presented in xylem sap when both *CYP735A1* and *CYP735A2* genes were knocked-out (Ko et al., 2014). Presumably disruption of two *CYP735A* genes depleted conversion of iP to tZ, consequently the overaccumulation of iP species in the xylem parenchyma cells resulted in their passive release into apoplastic space (xylem vessel). Alternatively, it is also possible for the existence of a low affinity exporter for iP-type species in root xylary cells. When high concentration of iPs accumulate, the transporter turns on. Molecular mechanism for iP systemic translocation needs to be further clarified.

## Transporters Involved in CK Signal Perception

The transported CKs, when reaching the target cells, will eventually meet their receptors either at the cell surface or inside the cells to trigger signaling cascade. The activity of CK transporters guides cellular and subcellular localization of CKs and affects ligand-receptor interactions. Fragmentary evidences indicate that both PUP and ENT family transporters participate in CK uptake, therefore, directly or indirectly affect CK cellular or subcellular distribution and signal perception. PUPs are a class of small, integral membrane proteins that exhibit influx activity to a broad range of nucleobases (Girke et al., 2014; Table 1). Similar to the discovery of ENTs, the recognition of PUPs also came from complementation assay by adopting a purine transport-deficient yeast mutant transformed with an Arabidopsis cDNA library. In the subsequent competition assays, besides adenine and other nucleobases, it was found that AtPUP1 also took up CKs kinetin and zeatin, meanwhile showed a lower activity to their ribosides (Gillissen et al., 2000). This discovery eventually led to the recognition of 23 PUP family members in Arabidopsis (Schwacke et al., 2003; Zürcher et al., 2016). So far AtPUP1, AtPUP2, and AtPUP14 have been demonstrated with direct CK uptake activity in yeast expression system, Arabidopsis mesophyll protoplasts, and/or microsomes derived from *Nicotiana benthamiana* transfected with *PUP* genes (Bürkle et al., 2003; Zürcher et al., 2016; Table 1). Interestingly, AtPUP1 and AtPUP2 behaved as the proton-gradient dependent symporter. Protonophore carbonyl cyanide *m*-chlorophenylhydrazone (CCCP) and H^+^-ATPase inhibitor significantly depleted their uptake activity to the radiolabeled tZ (Bürkle et al., 2003; Cedzich et al., 2008); whereas the activity of AtPUP14 is seemingly independent of proton-gradient potential but requests the presence of ATP (Zürcher et al., 2016). This phenomenon suggests that PUP members differ substantially in their biochemical properties. Moreover, *PUP* members *in planta* exhibit substantially different gene expression patterns, which infer their potential multifaceted physiological roles. For instance, *AtPUP1* is mainly expressed in the epithem of hydathodes and at the stigma surface of siliques (Figure 1), implicating its potential role in the retrieval of nucleobase derivatives in its expressing tissues (Bürkle et al., 2003). *AtPUP14* prevailed in all the examined tissues including seedlings, embryos and mesophyll protoplasts, where it displays the highest expression level among all other family members (Zürcher et al., 2016). In heart stage embryos, the expression of *AtPUP14* was inversely correlated to the cytokinin signaling readout. The reciprocal correlation led to a conclusion that AtPUP14, as a principal plasma membrane-resident protein, is able to import the active CK nucleobases into the cells consequently depleting apoplastic CK pools and inhibiting perception of CK signals by plasma membrane-localized receptors, by which, AtPUP14 modulates spatiotemporal CK sink patterns and influences plant morphogenesis (Zürcher and Muller, 2016; Zürcher et al., 2016). This hypothesis, however, evoked substantial argument in respect to whether the CK signaling initiation site occurs at plasma membrane or ER, since so far the experimentally defined CK receptors in different plant species appeared as the ER-localization (Romanov et al., 2006; Lomin et al., 2011, 2018). In addition, there are also some concerns about the binding affinity of PUPs versus the CK receptors to the ligands. Even if CK sensors do localize on the plant membrane, unless they have substantially lower ligand binding affinity than PUPs, it is not easy to explain the complete inhibition of CK signaling by the activity of AtPUP14 (Duran-Medina et al., 2017; Kang et al., 2017; Romanov et al., 2018). With these concerns, Romanov et al. (2018) argued that AtPUP14 or other PUP family members, instead of as the PM-localized proteins, might function at the ER membrane, where they pump CKs from ER lumen to the cytosol, thereby modulating CK signaling. Recently a rice PUP member OsPUP7 has been proved to localize at the ER and be involved in CK transport, although its flux pump direction remains ambiguous (Xiao et al., 2018), which seemingly aligns with Romanov et al’s argument.

In favor of CK signal perception primarily at the ER, Romanov et al proposed a model of multiple sites cellular CK signaling initiation and perception (Romanov et al., 2018), which integrates the importer activities of both PUP and ENT family proteins and differentiates the fate of tZ and tZR. The root-derived tZ that is transported through xylem to the particular region or cell types of the shoot directly interacts with the hypothetic plasma membrane-localized CK sensors, thereby, triggering hormonal signaling at the surface of cells. The established apoplastic CK pool can also be either degraded by the extracellular cytokinin oxidases, or transformed into the corresponding riboside by extracellular purine nucleoside phosphorylases, or imported into the cytoplasm by PUP transporters. The imported CK bases then could be either degraded or transformed into the inactive tZRP, and re-enter into the intracellular CK pool. On the other hand, the xylem-delivered tZR is taken up into the cells of the shoot via nucleoside importer ENTs. The imported tZRs in the cytosol could further undergo metabolic conversion by tZR kinase into tZRP, and by the activity of LOG enzymes release the biologically active form, free base of tZ.

While this multiple-site CK signal perception model integrates well with the experimental evidences on the ER-localization of CK sensors, the proposed transporter components call for more solid experimental confirmation. Primarily, the transport efficiency and specificity of ENTs to tZR is a matter of concern. The ENTs identified from Arabidopsis and rice exhibit quite broad substrate specificity and are apparently not optimal for the transport of tZR, since the affinity of the transporters to tZR (*K_m_* = 630∼660 μM) is substantially lower than that to iPR (*K_m_* = 17∼32 μM) or adenosine (*K_m_* = 3∼91 μM) (Hirose et al., 2005, 2008). Although it was reported that mutation of AtENT8 has suppressed the phenotypes of a plant with overproduction of CKs, and a loss-of-function of AtENT3 presented reduced CK uptake (Sun et al., 2005), compelling biological evidence for ENTs’ involvement in the tZR uptake is still missing.

## Future Perspectives

Short- and/or long-distance translocation of CKs are important components in CK biosynthesis, salvage, homeostasis, and signaling processes. Elucidating molecular mechanism of CK transport and identifying the related molecular factors of CK translocation have substantially deepened our understandings of the physiological roles that CKs play with in plant growth and development, and in plant-environmental interactions. Grafting experiments between *abcg14* mutant and WT have revealed that root-to-shoot transport of CKs is crucially required for the regulation of shoot growth and development (Ko et al., 2014). A recent study through disturbing root CK synthesis and translocation discovered that both tZ and tZR act as systemic signals traveling from root to shoot through xylem, differentially influencing leaf size and/or vegetative meristem activity (Osugi et al., 2017). Moreover, the long-distance translocated CKs also function as second messengers to signal the level of nutrient availability, particularly the soil nitrate level, to the shoot apical meristem, thereby mediating the organogenesis activity of shoot apical meristem in respect to the availability of soil mineral nutrients (Landrein et al., 2018). Aided with split-root system to uncouple local and systemic signaling, analysis of mutants *ipt3,5,7* and *abcg14* deficient in the root CK synthesis and translocation revealed that accumulation of the root-born tZ in shoots is an integral component of systemic signaling network of the root-shoot-root, which mediates the molecular and physiological responses of plants to nitrate heterogeneity in their roots, and controls gene expression in roots and shoots (Poitout et al., 2018). These studies exemplify the importance of unveiling CKs transport in understanding the physiological functions of this group of mobile hormonal molecules.

On the other hand, although a few transporter proteins have been implicated or partially evidenced in CK signal transport and distribution, the biological relevance of many putative CK transporter members require more detail and systematic exploration. PUP as well as ENT proteins are encoded by a multigene family *in planta*. The potential functional redundancy and their substrate promiscuity pose significant challenges for pinpointing their biochemical and biological functions in CK synthesis, intracellular traffic, cellular distribution and signal perception. To unequivocally elucidate the functions of ENTs and PUPs, it requires the integrated biochemical and biophysical approaches to precisely determine their influx or efflux carrier properties, substrate preferences, and subcellular localizations. Meanwhile, establishing higher order of mutant lines via conventional genetic cross and/or by CRISPR-Cas9 gene editing, together with the employment of high sensitive CK signal reporter system such as *TCSn::GFP* (Zürcher et al., 2013; Kirschner et al., 2018) could facilitate the determination and discrimination of biological roles of both PUP and ENT members in CK metabolism and signaling.

Recent study revealed that tZ and tZR transported through xylem flow exert distinct physiological roles for leaf development and apical meristem activity. The ratio of tZ and tZR in xylem flow and their delivery rate are modulated in response to the environmental conditions, which render different physiological consequence of shoot growth (Osugi et al., 2017). Currently it remains unclear if transporter proteins are involved in determining the ratio and delivery rate of root-born CK species in xylem flow. In the future, besides further tackling down additional physiological roles of ABCG14 in aerial tissues pertaining to the CK distribution, it is also intriguing to explore whether ABCG14, the primary transporter for xylem loading of tZ and tZR in root, coordinates with particular PUPs and/or ENTs in shoot to fine-tune the status and flow rate of different CK species in xylem sap and their differential cellular distribution, in response to plant growth and development cues and environmental stimuli.

## Author Contributions

C-JL wrote the manuscript. C-JL, YZ, and KZ prepared the data. All authors have read, edited, and agreed to the content.

## Conflict of Interest Statement

The authors declare that the research was conducted in the absence of any commercial or financial relationships that could be construed as a potential conflict of interest.
